# Totally Video-Assisted Thoracoscopic Surgery for Lung Transplantation: A Case Series

**DOI:** 10.3389/ti.2026.15873

**Published:** 2026-04-17

**Authors:** Qiang Pu, Xiaolong Zhang, Jian Zhou, Qian Yang, Xuehua Tu, Jianrong Hu, Ningying Ding, Jiao Chen, Jun Zeng, Yuchen Huang, Jiandong Mei, Lin Ma, Chenglin Guo, Dong Tian, Lunxu Liu

**Affiliations:** 1 Department of Thoracic Surgery and Institute of Thoracic Oncology, West China Hospital, Sichuan University, Chengdu, Sichuan, China; 2 Operating Room, Department of Anesthesiology, West China Hospital, West China School of Nursing, Sichuan University, Chengdu, Sichuan, China; 3 Department of Anesthesiology, West China Hospital, Sichuan University, Chengdu, Sichuan, China

**Keywords:** lung transplantation, minimally invasive surgery, video-assisted thoracoscopic surgery, case series, surgical technique

Dear Editors,

Lung transplantation remains the only effective treatment for end-stage lung disease. Traditionally, the Clam-Shell incision is the standard approach for bilateral sequential lung transplantation [1]; however, it requires a long incision and sternotomy, resulting in significant trauma [2] and extended recovery [3]. Accordingly, less invasive strategies have been explored, including bilateral anterolateral incisions without sternotomy [3], thoracoscopic-assisted techniques [4, 5], and robotic-assisted lung transplantation [6, 7]. Notably, most published experiences to date describe hybrid minimally invasive approaches, rather than totally video-assisted thoracoscopic surgery (VATS) as a standalone technique for lung transplantation.

In this context, we report our initial experience with totally VATS lung transplantation without rib retraction. Five consecutive patients with end-stage lung disease underwent this procedure at West China Hospital, Sichuan University, since December 2023. All operations were performed under VA-ECMO support. Baseline and clinical characteristics are summarized in Figure 1F.

**FIGURE 1:**
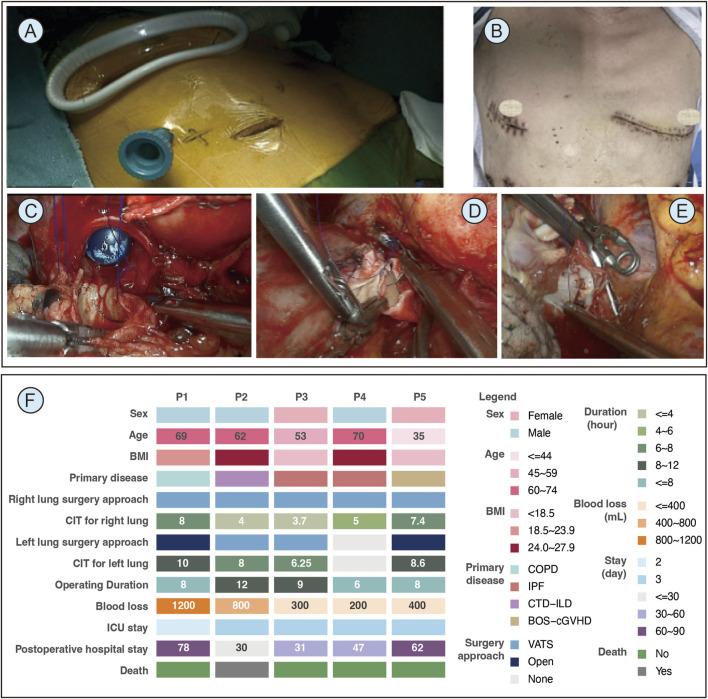
Surgical steps and patient outcomes of the totally video-assisted thoracoscopic surgery for lung transplantation. **(A)** The three-port incision design for totally VATS right lung transplantation. **(B)** Postoperative wound healing at the Patient 1 incision site; the right-side shows thoracoscopic incisions, while the left side shows open surgical incisions. **(C)** Right bronchial anastomosis. **(D)** Right pulmonary artery anastomosis. **(E)** Left atrial cuff anastomosis performed on the right side. **(F)** Waffle plot depicting patient characteristics as well as perioperative and long-term outcomes. Abbreviation: ICU, intensive care unit; CIT, cold ischemia time; BMI, body mass index; COPD, chronic obstructive pulmonary disease; IPF, idiopathic pulmonary fibrosis; CTD-ILD, connective tissue disease–related interstitial lung disease; BOS-cGVHD, bronchiolitis obliterans syndrome related to chronic graft-versus-host disease; VATS, video-assisted thoracoscopic surgery.

In our practice, we generally prioritize right-sided lung transplantation first. If the first-side procedure is excessively time-consuming, we convert to open thoracotomy for the contralateral side to avoid prolonged cold ischemia time. The surgical technique of totally VATS right lung transplantation is illustrated using Patient 1 as a representative case.

Patient was positioned supine with shoulders and back elevated to the nipple plane, arms abducted. A three-port incision design was used: an 8 cm main incision in the 4th intercostal space, a camera port in the 6th along the anterior axillary line, and an accessory port in the 6th along the midclavicular line (Figure 1A). The 8 cm incision was unnecessary for totally VATS resection and transplantation but required for donor lung implantation. The accessory and camera ports were interchangeable, with both used for chest tube placement.

The right pneumonectomy was performed using a single-direction thoracoscopic technique [8], proceeding anteriorly to posteriorly. The anterior apical branch of the pulmonary artery was addressed first, followed by the superior pulmonary vein, main pulmonary artery, inferior pulmonary vein, and right main bronchus. The vessels were stapled with an ETHICON ECR45W stapler. The main bronchus was dissected with an ultrasonic scalpel and scissors, then trimmed before anastomosis using scissors. A longitudinal pericardial incision anterior to the right phrenic nerve exposed the hilum and facilitated anastomoses. The incision was sutured and was brought out near the sternal edge of the incision, retracting the pericardium toward the anterior chest wall and suspending it. Then, an oval forceps was placed around the pericardial fold near the pulmonary veins, and the pericardium around the incision and veins was gradually separated to expose the left atrium.

The donor lung was inserted through the main incision, with an oval forceps through the accessory port to retract the apex and position the lung optimally. Anastomosis was performed in the same order as open surgery (Figures 1C–E). Pulmonary artery occlusion was achieved with a releasable Bulldog clamp (Aesculap, Inc., Center Valley, PA, USA) inserted through the accessory port. Left atrial occlusion was performed using an auricular appendage clamp. All anastomoses were performed using Prolene sutures with a continuous technique. The procedure began at the dorsocranial side, progressing downward to totally three-quarters of the circumference, then returning to finish the final quarter. Knotting was performed at the ventral midpoint. Suture traction was assisted using atraumatic forceps through the main or accessory ports. The anastomosis was primarily done under thoracoscopic guidance, with direct visualization via the main incision when necessary.

Perioperative and long-term clinical outcomes are detailed in Figure 1F. All cases maintained acceptable cold ischemia times and operative durations. No intraoperative emergency was observed. For postoperative complications, Patient 1 developed infections with multidrug-resistant *Pseudomonas aeruginosa*, *Candida* albicans, and *Corynebacterium striatum* 2 weeks postoperatively, requiring prolonged antimicrobial treatment, and was discharged on POD78, fully recovered and off supplemental oxygen. For long-term outcomes, Patient 2 died of gastrointestinal hemorrhage 6 months postoperatively, while the remaining recipients achieved successful recovery.

We preliminarily evaluated the potential benefits of totally VATS lung transplantation by comparing perioperative outcomes between the conventional open approach (n = 101) and totally VATS surgery (n = 5). Postoperative length of hospital stay was comparable between the open and VATS groups (41.70 ± 30.33 vs. 48.60 ± 20.22 days). No anastomotic fistula or stenosis was observed in the VATS group, whereas the rates in the open group were 3.96% and 2.97%, respectively. These observations should be interpreted with caution given the limited sample size.

Additionally, intraoperative parameters were compared within Patient 1, who underwent totally VATS transplantation on the right side and an anterolateral open approach on the left side (Figure 1B). Operative time was shorter for the VATS side (231 vs. 256 min), with reduced blood loss (400 vs. 800 mL). Postoperative incision-related pain, assessed by VAS, was also lower on the VATS side upon awakening (4 vs. 6).

Unlike previous “minimally invasive lung transplant” approaches involving mini thoracotomy [4, 5], the technique described herein employs a totally thoracoscopic method without rib spreading, minimizing chest wall disruption and promoting faster recovery.

Successful completion of thoracoscopic procedures relies on several key factors. First, a supine position is required for thoracoscopic pneumonectomy, with precise anatomical identification essential, particularly when freeing the left atrium to avoid injury. For anastomosis, the limited instrument angle due to the unexpanded incision may necessitate combining thoracoscopic guidance with direct visualization through the incision.

Patients with a large thoracic cavity, wide intercostal spaces, and a relatively small heart are generally more suitable candidates for VATS lung transplantation. In recipients with chronic obstructive pulmonary disease (COPD), anatomical characteristics associated with hyperinflated lungs may facilitate thoracoscopic manipulation. However, the presence of dense pleural adhesions can significantly limit the feasibility of a totally thoracoscopic approach.

Our findings suggest that VATS lung transplantation does not substantially prolong cold ischemia time or operative duration compared with conventional open surgery. Recent advances in donor lung preservation, such as 10 °C cold storage, or 4 °C–8 °C portable device for static hypothermic preservation may further enhance the feasibility of this minimally invasive approach [9], potentially enabling its application in more complex cases. Finally, Prospective comparative studies and long-term follow-up are needed to confirm its advantages over conventional thoracotomy.

## Data Availability

The original contributions presented in the study are included in the article/supplementary material, further inquiries can be directed to the corresponding author.

## References

[1] Patterson GA . Indications. Unilateral, Bilateral, Heart-Lung, and Lobar Transplant Procedures. Clin Chest Med (1997) 18(2):225–30. 10.1016/s0272-5231(05)70373-7 9187816

[2] Hernández-Hernández MA Sánchez-Moreno L Orizaola P Iturbe D Álvaréz C Fernández-Rozas S A Prospective Evaluation of Phrenic Nerve Injury After Lung Transplantation: Incidence, Risk Factors, and Analysis of the Surgical Procedure. J Heart Lung Transpl (2021) 41(1):50–60. 10.1016/j.healun.2021.09.013 34756781

[3] Marczin N Popov A-F Zych B Romano R Kiss R Sabashnikov A Outcomes of Minimally Invasive Lung Transplantation in a Single Centre: The Routine Approach for the Future or Do We Still Need Clamshell Incision? Interact Cardiovasc Thorac Surg (2016) 22(5):537–45. 10.1093/icvts/ivw004 26869662 PMC4892148

[4] Fischer S Strüber M Simon AR Anssar M Wilhelmi M Leyh RG Video-Assisted Minimally Invasive Approach in Clinical Bilateral Lung Transplantation. The J Thorac Cardiovasc Surg (2001) 122(6):1196–8. 10.1067/mtc.2001.118272 11726896

[5] Thomas J Chen Q Malas J Barnes D Roach A Peiris A Impact of Minimally Invasive Lung Transplantation on Early Outcomes and Analgesia Use: A Matched Cohort Study. J Heart Lung Transpl (2024) 43(8):1358–66. 10.1016/j.healun.2024.01.014 38310997 PMC11972168

[6] Emerson D Catarino P Rampolla R Chikwe J Megna D . Robotic-Assisted Lung Transplantation: First in Man. J Heart Lung Transpl (2023) 43(1):158–61. 10.1016/j.healun.2023.09.019 37778524

[7] Emerson D Megna D Razavi AA DiChiacchio L Malas J Rampolla R Robotic Lung Transplantation: Feasibility, Initial Experience, and 3-Year Outcomes. The Ann Thorac Surg (2025) 119(5):1107–16. 10.1016/j.athoracsur.2025.03.005 40118360 PMC13132875

[8] Liu L Che G Pu Q Ma L Wu Y Kan Q A New Concept of Endoscopic Lung Cancer Resection: Single-Direction Thoracoscopic Lobectomy. Surg Oncol (2010) 19(2):e71–e77. 10.1016/j.suronc.2009.04.005 19500971

[9] Cypel M Hötzenecker K Campo-Cañaveral de la Cruz J Kukreja J Suarez E Smith M Lungs Preserved on Ice or in a Refrigerator? Prolonged Static Lung Storage at 10° C. The Ann Thorac Surg (2023) 115(5):1095–7. 10.1016/j.athoracsur.2022.12.047 36787842

